# Editorial for “New Advances in Neurosurgery: Clinical Diagnosis, Treatment and Prognosis”

**DOI:** 10.3390/diagnostics15162000

**Published:** 2025-08-10

**Authors:** Janez Ravnik

**Affiliations:** Department of Neurosurgery, University Medical Centre Maribor, 2000 Maribor, Slovenia; janez.ravnik@ukc-mb.si

Neurosurgery represents one of the most rapidly evolving disciplines in modern medicine. Technological innovations, novel imaging modalities, and emerging treatment strategies have significantly enhanced both the understanding and management of disorders affecting the brain, spine, and peripheral nervous system.

The routine application of high-resolution preoperative imaging, intraoperative neuronavigation, fluorescence-guided resection, intraoperative imaging, and neurophysiological monitoring have markedly improved the safety and efficacy of brain tumor surgery [1]. These advancements have facilitated more extensive resections of malignant primary brain tumors [2]. In particular, the integration of 5-aminolevulinic acid (5-ALA) and intraoperative imaging have become the standard of care in many neurosurgical centers. Furthermore, endoscopic endonasal approaches have seen considerable refinement in recent years [3], offering enhanced safety profiles and becoming the preferred approach for an increasing number of tumors located at or near the skull base [3].

Endovascular techniques have undergone transformative progress, now allowing for the minimally invasive management of a wide range of cerebrovascular and spinal vascular pathologies [4]. Despite these advances, traditional open microsurgical approaches continue to play a critical role, particularly in complex cases, supported by minimally invasive strategies, novel instrumentation, and detailed patient-specific anatomical planning [5].

The combination of sophisticated imaging technologies, precise neuronavigation, intraoperative visualization, and refined surgical tools has enabled neurosurgical interventions to become increasingly less invasive [6]. Contemporary procedures often require only a small cranial opening and utilize narrow operative corridors to access deep-seated intracranial and spinal lesions, reducing patient morbidity and enhancing recovery.

Pediatric neurosurgery continues to pose unique challenges due to the complexity and heterogeneity of childhood neurological disorders. Significant strides have been made in understanding the biological underpinnings of various pediatric diseases. Nonetheless, treatment outcomes for certain conditions, particularly malignant pediatric brain tumors, remain suboptimal, underscoring the need for continued research and innovation [7].

Advanced multimodal monitoring has become increasingly widespread in the management of TBI. Given that brain trauma remains a leading cause of morbidity and mortality worldwide, optimal outcomes are best achieved through care delivered in specialized, high-volume centers equipped with the necessary expertise and infrastructure [8].

In this Special Issue, we present a range of innovative solutions across various neurosurgical domains. The benefits of minimally invasive surgery in the treatment of spontaneous intracerebral hematomas are highlighted, alongside advancements in endoscopic endonasal approaches detailed in two contributions. We introduce telementoring as a promising strategy to enhance surgical outcomes in low-volume centers. Within pediatric neurosurgery, we explore surgical outcomes for sagittal craniosynostosis and occult spinal dysraphisms, demonstrating that specialized care in dedicated centers yields superior results. A comprehensive retrospective study spanning more than two decades offers insights into the management of spinal dural arteriovenous fistulas, emphasizing the predictive value of MRI findings on neurological outcomes. Two studies showcase the critical role of advanced imaging in optimizing treatment strategies for glial tumors. Additionally, two case reports illustrate the challenges encountered in the management of vestibular and cervical schwannomas. Another large-scale study investigates seizure management strategies during carotid endarterectomy. Finally, a detailed anatomical study provides novel insights into vascular injuries sustained in firearm-related brain trauma.

Although outcomes for benign cranial tumors have improved significantly, the management of malignant brain neoplasms, particularly primary gliomas, remains a formidable challenge. The limitations of conventional microsurgical resection underscore the need for a dual approach: continued refinement of surgical technologies and a deeper understanding of the molecular and biological mechanisms driving disease progression. A similar paradigm applies to vascular neurosurgery. While endovascular interventions have become safer and more effective, the overall outcomes of many acute vascular pathologies remain unsatisfactory. For instance, an aneurysmal subarachnoid hemorrhage continues to carry a high mortality and morbidity burden. Even with timely aneurysm exclusion, there is a critical need for better strategies in the post-rupture management phase.

Neurosurgery is increasingly embracing a personalized medicine approach. Tailoring treatment strategies to individual patients’ anatomy, pathology, biological disease behavior, and personal preferences, while leveraging technical innovations, allows for the formulation of individualized therapeutic plans aimed at achieving optimal outcomes. The integration of artificial intelligence is anticipated to significantly enhance future management strategies [9].
